# A case of severe ARIA with multiple infarctions and extensive microbleeds following lecanemab administration

**DOI:** 10.1111/psyg.13231

**Published:** 2024-12-18

**Authors:** Akiko Yamazaki, Tetsuro Sekine, Shiro Takahashi, Koji Sohara, Masanori Sakamaki, Takehiko Nagao, Kazumi Kimura, Masahiro Mishina

**Affiliations:** ^1^ Dementia‐Related Disease Medical Centre Nippon Medical School Musashi Kosugi Hospital Kawasaki Japan; ^2^ Department of Neurology Nippon Medical School Tokyo Japan; ^3^ Department of Radiology Nippon Medical School Musashi Kosugi Hospital Kawasaki Japan; ^4^ Department of Neurology Nippon Medical School Musashi Kosugi Hospital Kawasaki Japan; ^5^ Department of Radiology Nippon Medical School Hospital Tokyo Japan; ^6^ Department of Neurology Tokyo Rosai Hospital Tokyo Japan

**Keywords:** amyloid‐related imaging abnormalities, anti‐amyloid β antibody, infarctions, lecanemab, microbleeds

## INTRODUCTION

Alzheimer's disease (AD), characterised pathologically by the presence of amyloid‐beta (Aβ) protein plaques, is the leading cause of dementia worldwide. Currently, lecanemab, an anti‐Aβ antibody, has been approved in many countries for the treatment of mild cognitive impairment and mild dementia caused by AD. However, it is associated with specific adverse events including immune‐mediated clearance, which carries the risk of vasogenic oedema and intracerebral haemorrhage, collectively referred to as amyloid‐related imaging abnormalities (ARIA). ARIA cases can be subdivided into ARIA with oedema or effusion (ARIA‐E), and ARIA with cerebral microhaemorrhage, cerebral macrohaemorrhage, or superficial siderosis (ARIA‐H). Previous studies have shown that even in severe cases of ARIA‐H, the number of microbleeds is limited to a few dozen.^1^ In addition, there have been no prior reports of the association of ARIA‐E with microinfarctions. Herein, we report a patient who developed numerous microbleeds, cerebral microinfarctions, and ARIA‐E after lecanemab administration.

## CASE PRESENTATION

**Figure 1:**
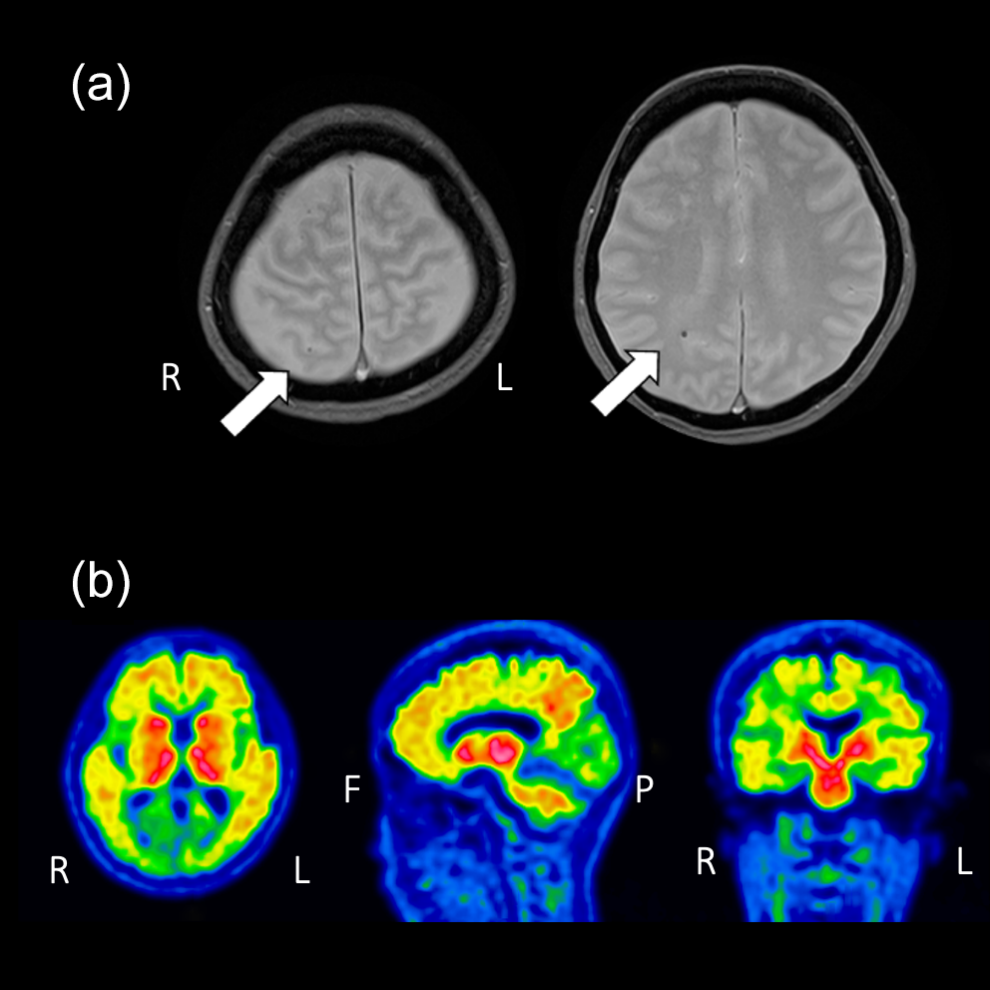
Neuroimaging results pre‐lecanemab/amyloid‐related imaging abnormalities (ARIA). Two microbleeds are identified using T_2_* magnetic resonance imaging (MRI) (a). Positron emission tomography imaging using ^18^F‐flutemetamol showed amyloid β plaque deposition in the frontal, lateral temporal and parietal lobe, posterior cingulate gyri, praecuneus, and striatum (b). The white arrows show microbleeds.

The patient was a 57‐year‐old married woman with 16 years of education. Her father was diagnosed with dementia in his 60s, and her mother in her 50s. She began experiencing mild memory symptoms at the age of 53. The patient first visited the memory clinic at our hospital at the age of 57 years. She was not taking any regular medications, including antihypertensives or antithrombotics. Her Mini‐Mental State Examination (MMSE) score was 26/30 and her score on the Japanese version of the Montreal Cognitive Assessment (MoCA) was 17/30, indicating disorientation and short‐term memory impairment, affecting daily life. Magnetic resonance imaging (MRI) of the head revealed mild atrophy of the left hippocampus and parahippocampal gyrus, along with two microbleeds (Fig. 1a). Positron emission tomography imaging using ^18^F‐flutemetamol revealed amyloid plaque deposition in the frontal, lateral temporal and parietal lobes, posterior cingulate gyrus, praecuneus, and striatum (Fig. 1b). Based on these findings, the patient was diagnosed with early‐onset AD. In Japan, genetic testing is not covered by national health insurance; therefore, it is not routinely performed in clinical practice. As genetic counselling was not available at our hospital, the apolipoprotein E (*APOE*) genotype was not assessed before receiving lecanemab. The patient met the eligibility criteria for lecanemab according to the Japanese guidelines for optimal use. She expressed a wish to undergo treatment with lecanemab, and thereafter started receiving 10 mg/kg lecanemab twice a month. By the third dose of lecanemab, she developed a fever, but did not use antipyretics. About 6 h after the fourth dose, she developed left hemiparesis, confusion, and urinary incontinence. She visited our hospital 2 days after the fourth dose.

**Figure 2:**
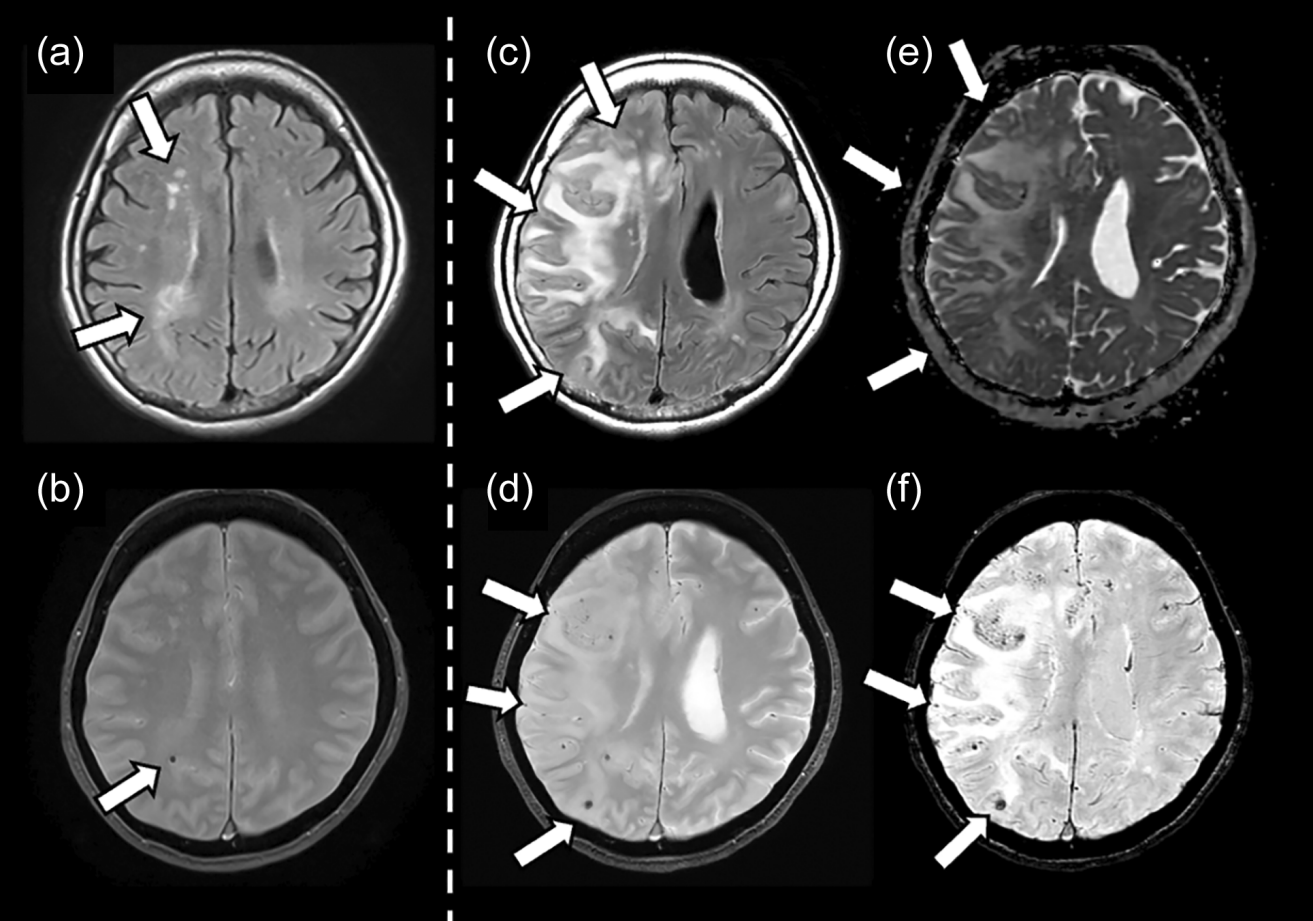
Magnetic resonance imaging (MRI) prior to treatment, and changes following treatment with lecanemab. Baseline MRI showing only subtle white matter hyperintensity on fluid‐attenuated inversion recovery (FLAIR). The white arrows show subtle white matter hyperintensity on FLAIR (a) and only two microbleeds on T_2_* weighted image (WI) (one of the two microbleeds is indicated by arrows in b, the white arrow shows microbleed). MRI taken 6 days following symptom onset showing extensive high‐signal areas in the white matter on FLAIR. The white arrows show extensive high‐signal areas in the white matter on FLAIR (c) and extensive microbleeds along the cortex and subcortical white matter on T_2_* WI. The white arrows show extensive microbleeds along the cortex and subcortical white matter on T2*WI (d). Consequently, the patient was diagnosed with severe amyloid‐related imaging abnormalities (ARIA) (E) and ARIA with haemorrhage (ARIA‐H) infections. The apparent diffusion coefficient map indicates increased diffusion. The white arrows indicate the presence of vasogeniv edema (e), indicating the presence of vasogenic oedema. Susceptibility‐weighted imaging (SWI), revealed numerous small microbleeds along the cortex. The white arrow show indicate new microbleeds (f), with more than 100 new microbleeds appearing throughout the brain. Despite numerous microbleeds, imaging findings did not indicate any superficial siderosis (data not shown).

**Figure 3:**
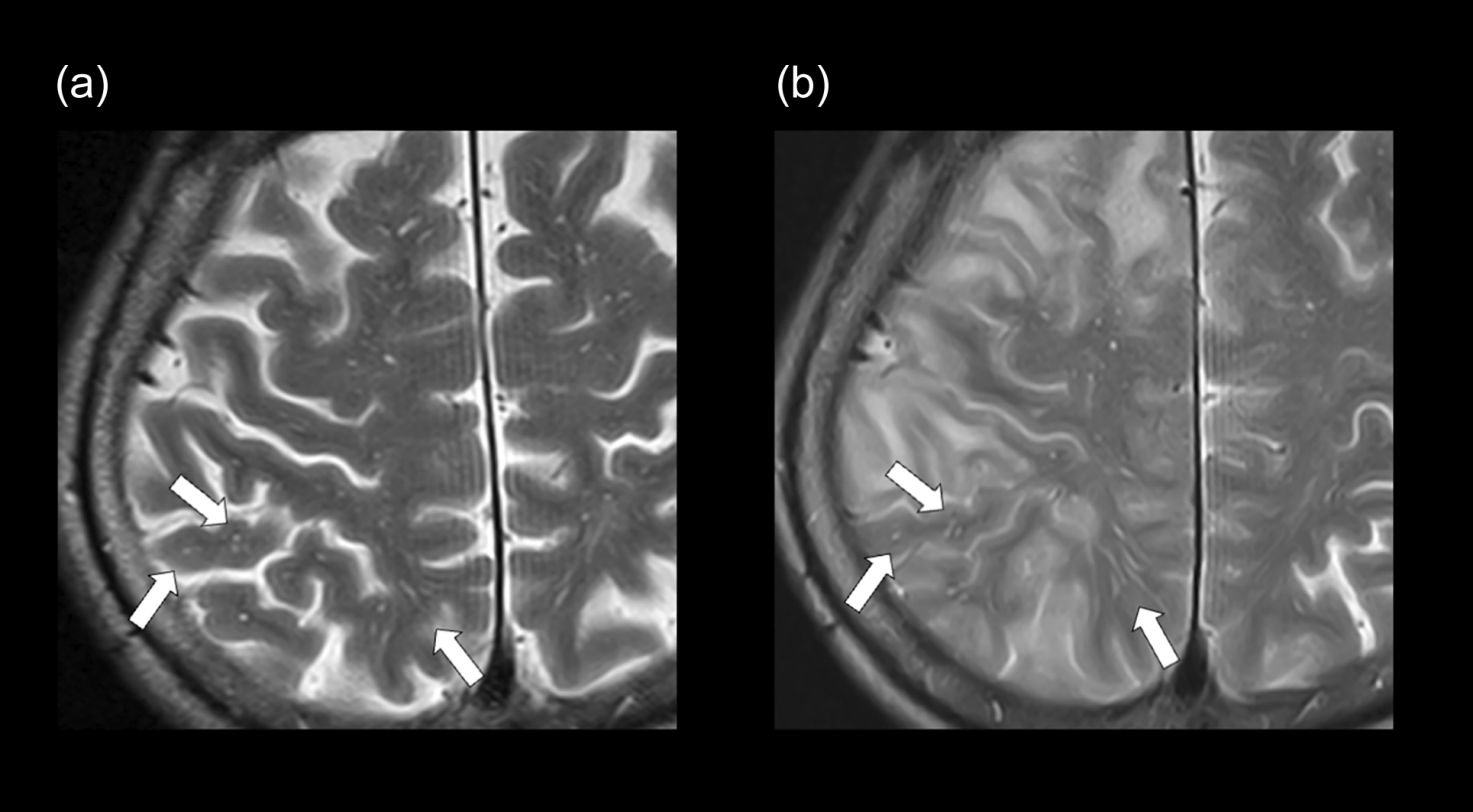
Changes in perivascular spaces before and after lecanemab treatment. Baseline T2 weighted image (WI) showing dilation of the perivascular space, thought to reflect impaired glymphatic drainage (a). At the onset of amyloid‐related imaging abnormalities (ARIA), the dilation of the perivascular space worsened despite swelling of the perivascular space (b). The white arrows show dilation of the perivascular space.

**Figure 4:**
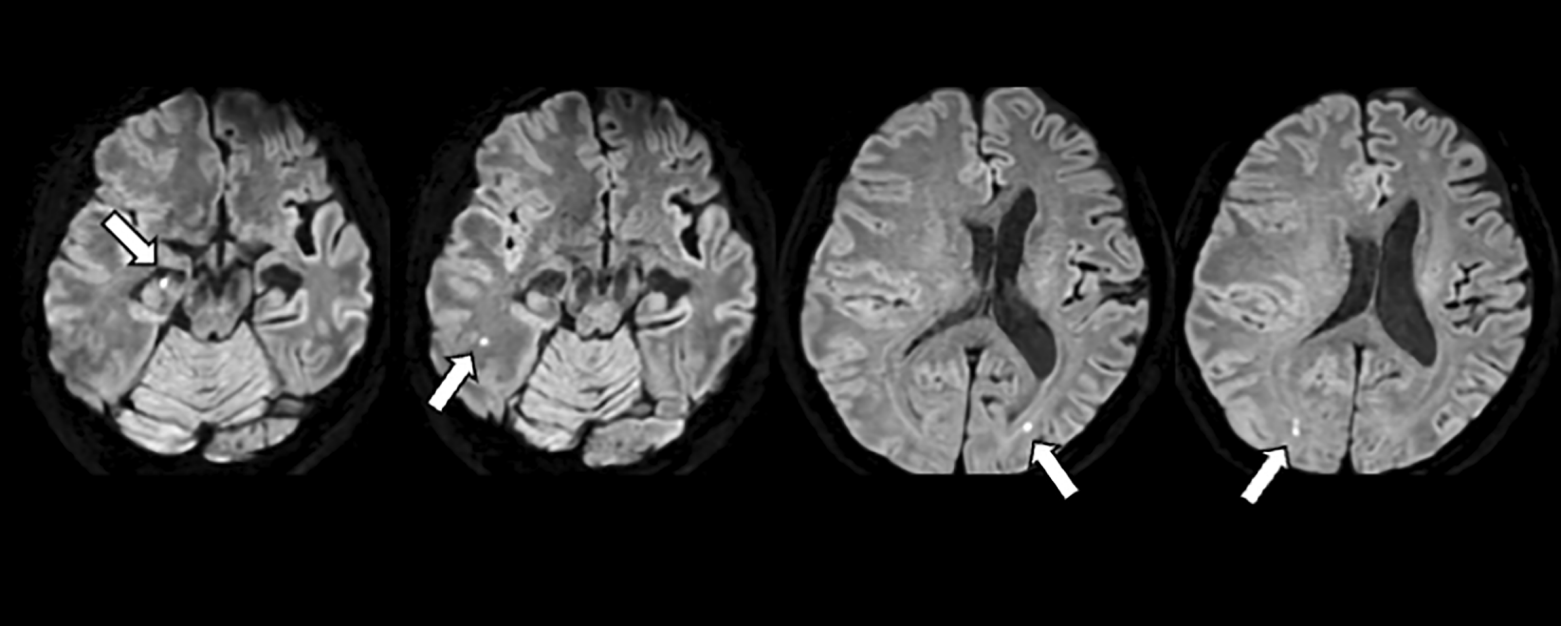
Multiple microinfarctions following lecanemab treatment. Thin‐slice diffusion‐weighted image (DWI) (slice thickness = 2 mm with gaplessness) performed 6 days following symptom onset revealed numerous punctate high‐signal areas. The white arrows show microinfarctions.

**Figure 5:**
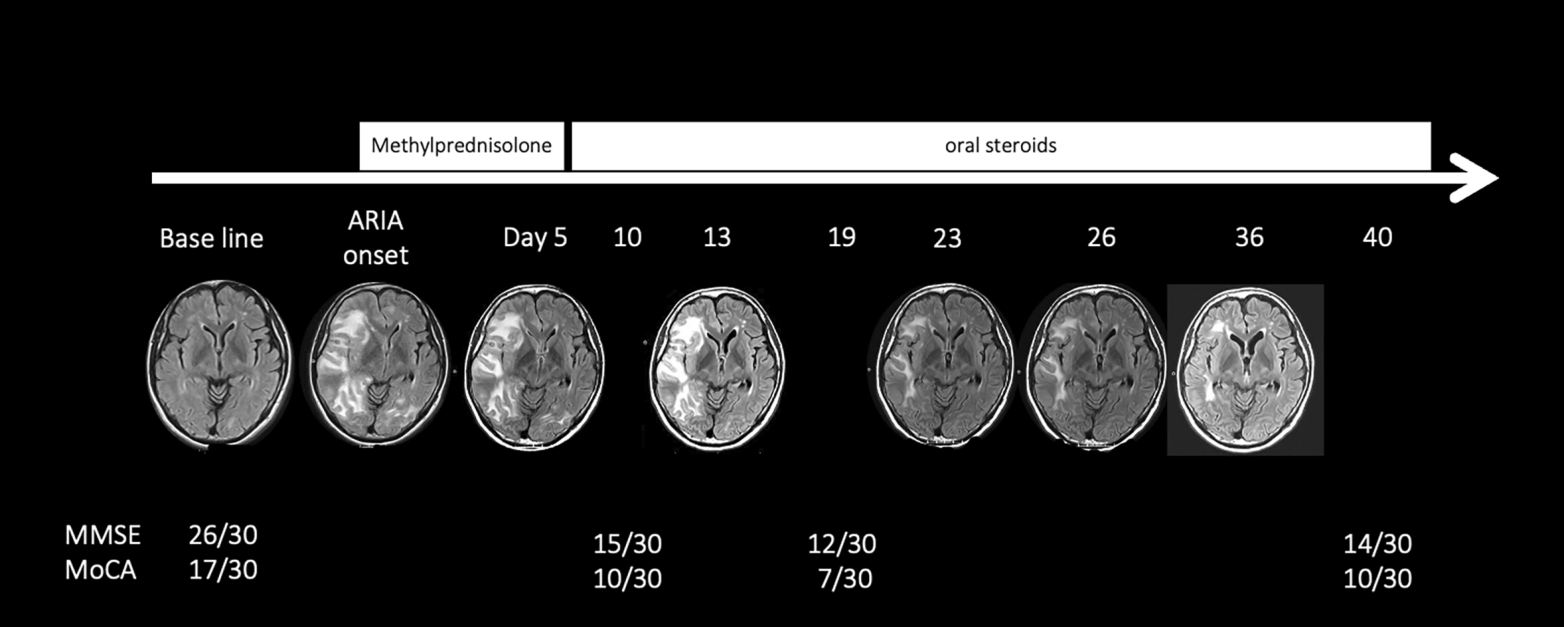
Temporal changes in amyloid‐related imaging abnormalities (ARIA) onset: Imaging improvements and cognitive function progression following steroid treatment.

At presentation, her symptoms included confusion, headache, and left hemiparesis. However, she was able to walk with assistance. On MRI, under the same baseline conditions, the number of microbleeds increased to 18, primarily appearing along the frontal and parietal cortices, and were more prominent on the right side. On susceptibility‐weighted imaging (SWI), this number increased to more than 100 (Fig. 2b,d,f). Multiple microinfarctions were observed in the temporal and occipital lobes of the cerebral cortex, as well as in the subcortical white matter (Fig. 3). Interestingly, despite progression of cerebral oedema, the perivascular spaces had expanded compared to baseline (Fig. 4). The patient was diagnosed with severe ARIA‐E and ARIA‐H, and was subsequently admitted to the hospital. She received intravenous methylprednisolone (1000 mg) for 5 days, followed by the initiation of oral steroid therapy. She was subsequently started on oral prednisolone (PSL) at a dose of 1 mg/kg body weight, which was tapered weekly; by the 29th day of illness, the dose was reduced to 20 mg. By the end of treatment, her headaches, left hemiparesis, and confusion had resolved, and follow‐up MRI revealed a significant improvement in ARIA‐E (Fig. 5). On the 10th day of treatment, her MMSE and MoCA scores were 15/30 and 10/30, respectively.

The patient was discharged and began outpatient treatment when the dose of oral steroids was reduced to 20 mg PSL. On the 40th day of the illness, the patients' MMSE and MoCA scores were 14/30 and 11/30, respectively. Following the development of ARIA, lecanemab treatment was discontinued. The patient will continue to be monitored at a facility specialising in dementia care.

## DISCUSSION

The current case met the diagnostic criteria for both severe ARIA‐E (fluid‐attenuated inversion recovery hyperintensity >10 cm with associated swelling and sulcal effacement) and ARIA‐H (10 or more new incident microhaemorrhages and >2 areas of superficial siderosis).

This case has four notable characteristics. First, the patient developed multiple cerebral microinfarctions due to ARIA. A PubMed search conducted using the search terms ‘(Amyloid‐Related Imaging Abnormalities) AND (infarction OR stroke)’ on August 12, 2024, yielded no prior reports of cerebral infarctions occurring as a result of ARIA. ARIA has been noted to present with similarities to cerebral amyloid angiopathy‐related inflammation (CAA‐ri).^2^ It has further been reported that cerebral infarctions occur in one of the severe subtypes of CAA‐ri, which is angiitis.^3^ It is possible that the present case showed similar aberrations of the microvasculature. Our experience with this case indicates that thin‐slice diffusion‐weighted image sequences should be included when ARIA is suspected.

The second unique characteristic was the presence of >100 microbleeds. Previous imaging reviews of ARIA‐H have reported a range of microbleeds, typically in the order of dozens, making the imaging findings of this case unique.^4^ Compared to one case report in which microbleeds were observed in detail using 7 Tesla‐MRI following the onset of ARIA, the microbleeds in this case are clearly numerous. Despite the presence of numerous microbleeds, the absence of superficial siderosis was a characteristic feature of this case, which may reflect the presence of vasculitis associated with local inflammatory responses rather than leakage of blood components due to vascular permeability.

Third, the patient exhibited severe changes sufficient to cause a midline shift. Most cases of ARIA treated with lecanemab are asymptomatic, with rare fatalities. Although the patient showed no severe clinical deterioration, the presence of a midline shift posed a risk of brain herniation in the current case, necessitating admission to the intensive care unit. Following hospitalisation, treatment with methylprednisolone, which is the recommended treatment for severe ARIA, was initiated, and oedema showed a tendency to decrease within a month. The headache and left hemiparesis resolved. The patient's cognitive function gradually improved.

Fourth, despite ARIA‐E‐associated brain swelling, perivascular space expansion was observed. This may indicate impaired clearance, specifically delayed excretion of Aβ in these regions, potentially burdening the glymphatic drainage system due to the removal of dissolved Aβ following treatment.^5^ This is thought to be one of the causes of ARIA development in the current case.

## CONCLUSION

Herein, we reported a case of ARIA with multiple unprecedented imaging features, with a focus on the mechanisms of vasculitis and the burden on the perivascular space. These imaging findings are helpful in understanding the mechanisms underlying the onset of ARIA. The cause of the ARIA in this case was unclear. It is possible that this patient was relatively young. In this case, unfortunately, *APOE* genotype was not measured, but we suspect that the patient should have an *APOE* ε4 homozygote. Further research is required to prevent the occurrence of ARIA in patients with AD treated with lecanemab.

## DISCLOSURE

Dr. Mishina, Masahiro, a co‐author of this article, is an Editorial Board member of *Psychogeriatrics*. The authors declare no conflict of interests for this article.

## ETHICS STATEMENT

Informed consent was obtained from the patient for the publication of this case report.

## Data Availability

Data sharing not applicable to this article as no datasets were generated or analysed during the current study.
